# Bioactive Sesterterpenes and Triterpenes from Marine Sponges: Occurrence and Pharmacological Significance

**DOI:** 10.3390/md8020313

**Published:** 2010-02-23

**Authors:** Sherif S. Ebada, WenHan Lin, Peter Proksch

**Affiliations:** 1 Institute of Pharmaceutical Biology and Biotechnology, Heinrich-Heine University, Universitaetsstrasse 1, D-40225 Duesseldorf, Germany; 2 State Key Laboratory of Natural and Biomimetic Drugs, Peking University, Beijing 100083, China; E-Mail: whlin@bjmu.edu.cn; 3 Department of Pharmacognosy and Phytochemistry, Faculty of Pharmacy, Ain-Shams University, Abbasia, Cairo, Egypt

**Keywords:** sesterterpenoids, triterpenoids, marine sponges

## Abstract

Marine ecosystems (>70% of the planet’s surface) comprise a continuous resource of immeasurable biological activities and immense chemical entities. This diversity has provided a unique source of chemical compounds with potential bioactivities that could lead to potential new drug candidates. Many marine-living organisms are soft bodied and/or sessile. Consequently, they have developed toxic secondary metabolites or obtained them from microorganisms to defend themselves against predators [1]. For the last 30–40 years, marine invertebrates have been an attractive research topic for scientists all over the world. A relatively small number of marine plants, animals and microbes have yielded more than 15,000 natural products including numerous compounds with potential pharmaceutical potential. Some of these have already been launched on the pharmaceutical market such as Prialt^®^ (ziconotide; potent analgesic) and Yondelis^®^ (trabectedin or ET-743; antitumor) while others have entered clinical trials, e.g., alpidin and kahalalide F. Amongst the vast array of marine natural products, the terpenoids are one of the more commonly reported and discovered to date. Sesterterpenoids (C_25_) and triterpenoids (C_30_) are of frequent occurrence, particularly in marine sponges, and they show prominent bioactivities. In this review, we survey sesterterpenoids and triterpenoids obtained from marine sponges and highlight their bioactivities.

## 1. Introduction

Terpenes include primary and secondary metabolites, all biosynthesized from the five carbon isoprene building units [2]. Structural modification of these isoprene units leads a massively diverse range of derivatives with a wide array of chemical structures and biological properties. While higher plants’ terpenoids were already studied and ethnopharmacologically rationalized centuries ago, those from marine counterparts were not explored until the first half of the 20^th^ century.

Steroidal terpenoids were the first marine isoprenes to be discovered by Bergmann during the 1930s–1940s, particularly sterols that were obtained from various marine macroorganisms [3]. Secondary metabolites, including terpenes, play an important ecological role in marine organisms. Being sessile and soft bodied, marine organisms face a harsh competition for space, reproduction, maintenance of an unfouled surface and deterrence of predation [4]. Therefore, marine organisms have developed bioactive secondary metabolites as a potential defensive means against competitors and/or predators [1]. These compounds are rapidly diluted after being released into the water and hence have to be of outstanding potency to retain their efficacy. These bioactivity(ies) proved appealing for chemical ecologists as well as for pharmacologists in their search for new drugs to treat or cure serious ailments such as inflammatory, infectious and cancerous diseases.

Marine terpenoids dominate much of the literature expression with a huge number of derivatives having been obtained from marine resources. It seems pointless to compile a review that includes all major classes of marine terpenoids. Therefore, in this review we concentrate on two major classes of marine isoprenes from sponges, namely the sesterterpenoids (C_25_) and triterpenoids (C_30_) with particular attention placed on their biological activities.

Marine triterpenoids were the first terpenoids reported from marine resources and since then a vast array of derivatives have been documented. In this review, we cover steroidal saponins and isomalabaricane triterpenoids. In addition, marine sponges have been identified as one of the prime resources of sesterterpenes and hence we also survey this class of marine terpenoids.

## 2. Sesterterpenes (C_25_)

Manoalide (**1**) is the parent compound of a series of marine sponge metabolites belonging to the sesterterpene class. Manoalide was first reported in 1980 by Scheuer from the marine sponge *Luffariella variabilis* (class Demospongiae; order Dictyoceratida; family Thorectidae) collected in Palau [5] with activity as an antibiotic against *Streptomyces pyogenes* and *Staphylococcus aureus*.

**Figure:**
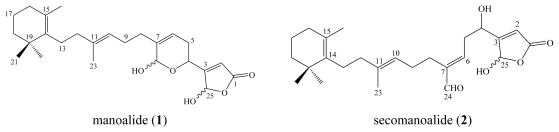


One year later, Scheuer reported three additional related metabolites from the same Palauan sponge, namely secomanoalide (**2**), (*E*)-neomanoalide (**3**) and (*Z*)-neomanoalide (**4**) [6]. All three compounds, as well as the parent compound (**1**), displayed antibacterial activity against Gram positive bacteria (*Staphylococcus aureus* and *Bacillus subtilis*) but were inactive against *Escherichia coli*, *Pseudomonas aeruginosa* and *Candida albicans* [6].

**Figure:**
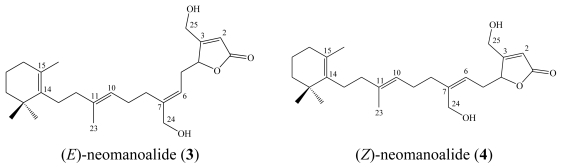


Later, marine sponges belonging to the family Thorectidae, including species of the genera *Luffariella* [7–19], *Hyrtios* [20,21], *Thorectandra* [22], *Cacospongia* [23,24], *Fasciospongia* [25–28], *Acanthodendrilla* [29] and *Aplysinopsis* [30], were also found to be rich sources of novel bioactive sesterterpenoids related to manoalide.

Manoalide was further investigated and found to be a potent inhibitor of phospholipase A_2_ (PLA_2_) [31–38]. Subsequently, many structurally related metabolites with PLA_2_ inhibitory activity were also reported [8, 39–45]. PLA_2_ is an enzyme that specifically catalyzes the hydrolysis of phospholipids at the *S_N_-*2 position to produce a lysophospholipid and arachidonic acid, which in turn provides the substrate for proinflammatory mediators such as leukotrienes, prostaglandins and thromboxanes, collectively known as the eicosanoids [41]. Since manoalide revealed an irreversible inhibition of phospholipase A_2_ (PLA_2_) [33], the structure-activity relationships (SAR) of this compound attracted scientific interests to study and to understand both PLA_2_ function and mechanism of action in the whole cell. Therefore, several studies were successfully performed to determine the contributions of the various functional groups incorporated in **1** and its analogs, such as the *γ*-hydroxybutenolide, *α*-hydroxydihydropyran and trimethylcyclohexenyl ring systems, to the efficacy as PLA_2_ inhibitors [36,41,45]. These studies indicated that (1) the existence of the hemiacetal in the *α*-hydroxydihydropyran ring is crucial for irreversible binding, (2) the *γ*-hydroxybutenolide ring is involved in the initial interaction with PLA_2_ and (3) the hydrophobic nature of the trimethylcyclohexenyl ring system allows non-bonded interactions with the enzyme that enhances the potency of these analogs. These studies suggested that the closed ring form of manoalide is the predominant molecular moiety that accounts for the selective and potent inhibition of PLA_2_ [36].

Manoalide analogs also exhibited other bioactivities including molluscicidal [10], cytotoxicity [13,14,16,20,23,26,29,30,47–49], inhibitory activity of Cdc25 phosphatase [46], nicotinic antagonistic activity [12] and fish deterrent properties [26,49]. Therefore, chemical synthesis and derivatization of manoalide attracted much interest leading to a better understanding of the structure activity relationships (SAR) and/or for the plausible mechanism of action [35,38–40,43,44,50,51]. Manoalide (**1**) was licensed to Allergan Pharmaceuticals and reached phase II clinical trials as a topical antipsoriatic. Its development was, however, discontinued due to formulation problems. The compound is now commercially available as a biochemical standard tool to block the action of PLA_2_ [52].

Luffariellolide (**5**) is a sesterterpenoid analog of secomanoalide (**2**), which was first reported from a Palauan sponge *Luffariella* sp. [8]. Structurally, luffariellolide differed in having C-24 as methyl group instead of an aldehyde functionality as in **2** and it was obtained as the (*Z*) isomer as well.

**Figure:**
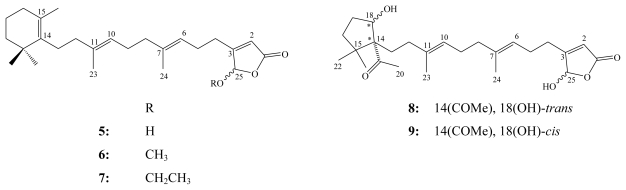


In contrast to the irreversible inhibitory action of manoalide (**1**) towards PLA_2_, luffariellolide (**5**) is a slightly less potent, but a partially reversible inhibitor. This meant that **5** became a more preferable anti-inflammatory agent for potential pharmacological investigation [8].

In addition to luffariellolide (**5**), its 25-*O*-methyl (**6**) and 25-*O*-ethyl derivatives (**7**), five related sesterterpenes, acantholides A–E, were obtained from the Indonesian sponge *Acanthodendrilla* sp. [29]. Acantholide D (**8**) and E (**9**) represent rare variants for the C_14_–C_20_ segment in this type of linear sesterterpenes in which they have the 1-acetylcyclopentan-5-ol moiety replacing the trimethylcyclohexenyl ring. Luffariellolide (**5**) and its 25-*O*-methyl congener (**6**), as well as acantholide E (**9**), were cytotoxic against the mouse lymphoma L5178Y cell line with IC_50_ values of 8.5, 1.8, and 16.8 μM, respectively. Interestingly, these results suggest that the 25-*O*-methyl group in **6** and the stereochemistry of 1-acetylcyclopentan-5-ol in **9** play an important role [29].

Luffariolides A–J represent a related group of sesterterpenoidal analogs, which have been obtained from different collections of the Okinawan marine sponge *Luffariella* sp. [13,14,16].

All luffariolides exhibited significant cytotoxicity against murine lymphoma L1210 cells with IC_50_ values ranging between 2.9–19.3 μM. Amongst them, luffariolides A (**10**, IC_50_ 2.9 μM), B (**11**, IC_50_ 3.23 μM), E (**12**, IC_50_ 3.0 μM) and F (**13**, IC_50_ 3.8 μM) were the most active ones [13,14,16].

**Figure:**
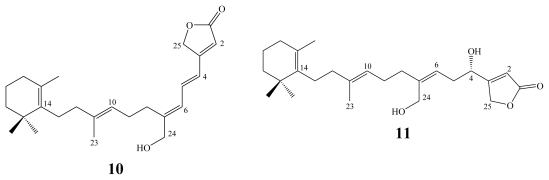


**Figure:**
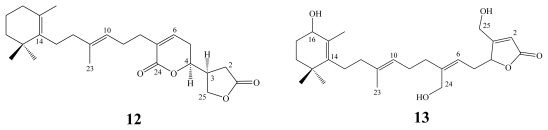


Luffariellins A (**14**) and B (**15**) [7] together with their respective 25-acetoxy derivatives (**18** and **19**) [18] were isolated from the marine sponge *Luffariella variabilis* collected off different locations in Palau and in Australia, whereas luffariellins C (**16**) and D (**17**) were obtained from the shell-less marine mollusc *Chromodoris funerea* collected from the Kaibakku lake shores in Palau [53].

**Figure:**
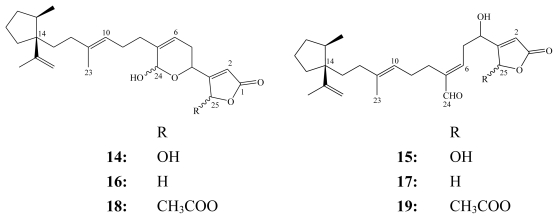


Luffariellins (**14**–**19**) are all characterized by the 1-isoproprenyl-2-methylcyclopentane ring system replacing the trimethylcyclohexenyl moiety in other manoalide analogs. Despite this discrepancy in chemical structure, luffariellins A (**14**) and B (**15**) retain identical functional groups as present in manoalide (**1**) and secomanoalide (**2**), respectively. Therefore, not surprisingly each respective pair was shown to have similar anti-inflammatory properties to **1** and **2** [7].

Luffarin metabolites comprise another group of compounds represented by 28 derivatives. 26 of them, luffarins A–Z, have been reported from the Australian marine sponge *Luffariella geometrica* [12], while the other two were obtained from the Adriatic Sea sponge *Fasciospongia cavernosa* [28]. Based on the chemical structures, luffarins have been classified into 14 bicyclic sesterterpenes, luffarins A–N; one bicyclic bisnorsesterterpene, luffarin O; one monocyclic sesterterpene, luffarin P; and six acyclic sesterterpenes, luffarin Q–V, in addition to four diterpenoidal derivatives, luffarin W–Z [12].

All luffarins were tested for antimicrobial activity against *Staphylococcus aureus*, *Micrococcus* sp., and *Saccharomyces cerevisiae*. Only luffarins C–F (**22**–**25**), K (**26**) and L (**27**) showed activity against both *S. aureus* and *Micrococcus* sp. [12], whereas luffarins A (**20**) and M (**28**) revealed only mild activity against the latter. Moreover, some luffarins were also found to be effective inhibitors of nicotinic receptors [12].

**Figure 1:**
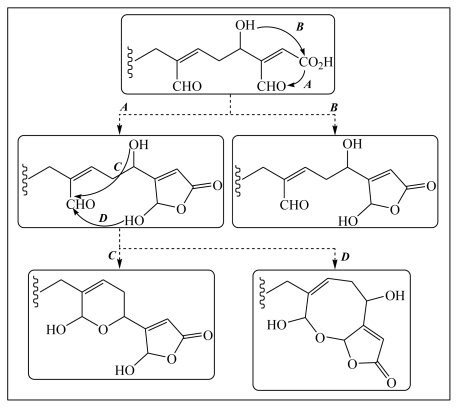
Postulated biosynthetic relationship between all known *Luffariella* metabolites [12].

Biosynthetically, a relationship could be recognized between the various luffarins as illustrated in Figure 1. Luffarins appear to belong to the same enantiomeric series as reported for manoalide-type marine natural products. It is also curious to note that no acyclic luffarins incorporated the hydroxylated butenolide functionality. Perhaps the most interesting luffarins from a biosynthetic point of view are luffarins B (**21**) and O (**21a**), which were the first examples of a hitherto unknown cyclization pattern in compounds of this class [12].

**Figure:**
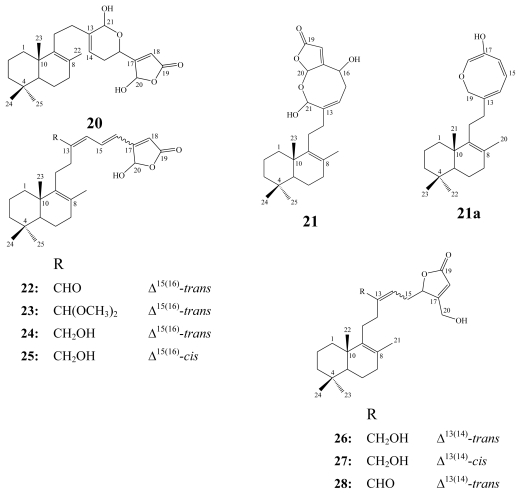


Another example of bicyclic sesterterpenes are thorectandrols A–E (**31**–**35**) that were isolated from a Palauan collection of the marine sponge *Thorectandra* sp. [47,48] together with the parent compounds of this group palauolide (**29**) and palauolol (**30**). Palauolide (**29**) was obtained first as an antimicrobial sesterterpene from a three sponge association collected in Palau [54]. While palauolol (**30**) was identified as an anti-inflammatory sesterterpene from the Palauan sponge *Fascaplysinopsis* sp. and chemically it was recognized as being a secondary alcohol that upon dehydration yields **29** [55].

**Figure:**
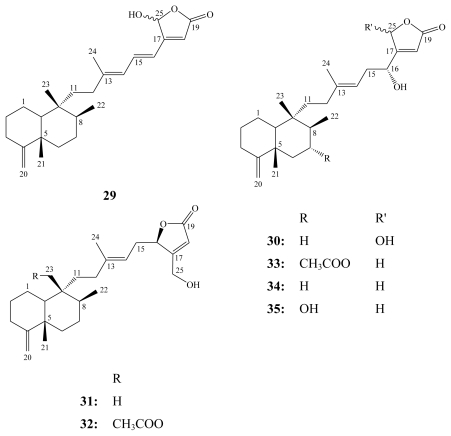


All thorectandrols (**31**–**35**) in addition to palauolide (**29**) and palauolol (**30**) were tested for antiproliferative activity against six to twelve human tumor cell lines depending on sample availability [48]. Palauolol (**30**) was active against all tested cell lines except A549 (non small lung cancer), with IC_50_ values in the range 1.2–1.7 μM, while palauolide (**29**) showed a diminished activity. On the other hand, thorectandrols A–E revealed only weak to no cytotoxicity against the tested cell lines (IC_50_’s 70–100 μM). While firm deductions on the structural requirements for activity were not possible, it appeared that the presence of both the hemiacetal lactone functionality and the 16-hydroxyl group in palauolol (**30**) enhanced cytotoxicity compared to palauolide (**29**) and other thorectandrols [48].

Cacospongionolides (**36**–**40**) were isolated from different collections of the marine sponge *Fasciospongia cavernosa* (=*Cacospongia mollior*) collected from the Mediterranean Sea [23,26,49,56]. Cacospongionolides A (**36**), B (**37**) and its 25-deoxy derivative (**38**) revealed a bicyclic sesterterpenoidal skeleton, resembling luffarins and thorectandrols, with the addition of a *γ*-hydroxybutenolide moiety. The other cacospongionolides C (**39**) and D (**40**) are acyclic diterpenoidal derivatives. Despite the structural relation with luffarins and thorectandrols, cacospongionolides (**36**–**38**) together with cacospongionolide D (**40**) exhibited significant cytotoxicity [23,26,49,56]. This notion suggested a possible relation between the presence of the *γ*-hydroxybutenolide moiety and the cytotoxicity.

**Figure:**
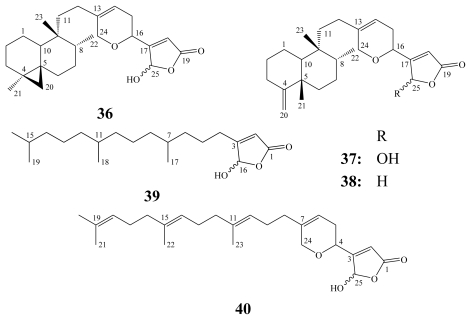


Petrosaspongiolides A (**41**) and B (**42**) were the first cheilantane sesterterpene lactones to be isolated from a New Caledonian sponge incorrectly assigned to the genus *Dactylospongia* [57] and then reassigned as a new genus and a new species: *Petrosaspongia nigra* (Bergquist 1995 sp. nov., class Demospongiae; order Dictyoceratida; family Spongidae) [58].

From another New Caledonian collection of the same sponge, 15 additional petrosaspongiolide congeners (C–R) were isolated [59,60].

**Figure:**
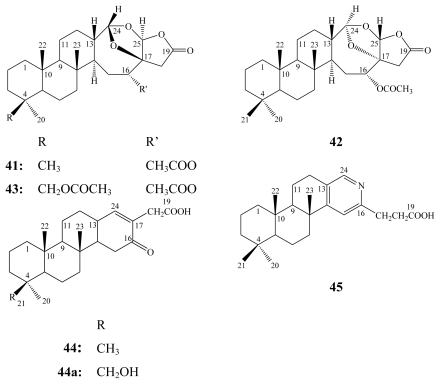


From the chloroform extract of another Dictyoceratida sponge of the genus *Spongia*, 21-hydroxy derivatives of petrosaspongiolides K (**44a**) and P (**48a**) were isolated in addition to four other pyridinium alkaloids named spongidines A–D (**51**–**54**) [61]. Spongidines were found to be structurally related to petrosaspongiolide L (**45**) particularly in the presence of pyridine ring.

**Figure:**
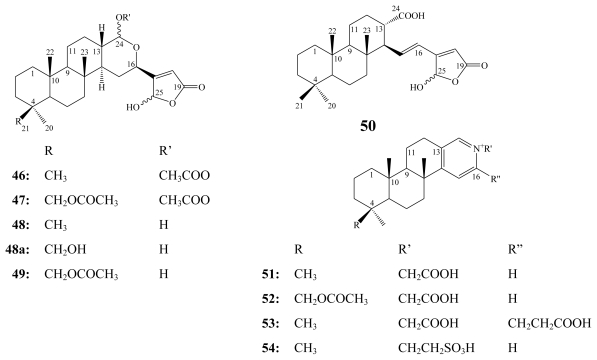


Petrosaspongiolides A–L were subjected to *in vitro* cytotoxicity assay against the human bronchopulmonary NSCLC-N6 carcinoma cell line. They revealed IC_50_ values ranging between 1.0–32.2 μM [59]. Petrosaspongiolides C (**43**) and K (**44**) exhibited the highest potency with IC_50_ values of 1.0 and 3.5 μM, respectively. However, petrosaspongiolides A (**41**) and B (**42**) were the least cytotoxic congeners *in vitro* with IC_50_ values of 28 and 32.2 μM, respectively, **41** inhibited tumoral proliferation *in vivo* at 20 mg/Kg without significant toxicity when tested on immunosuppressed rats carrying a bronchopulmonary tumor (NSCLC-N6) [59].

Petrosasponiolides M–R (**46**–**50**) revealed the presence of a *γ*-hydroxybutenolide moiety and a hemiacetal function. Due to these structural similarities to manoalide (**1**), petrosaspongiolides M–R have received special attention from the scientific community to study their inhibitory activity against PLA_2_ from different resources to point out their specificity. Two main groups of PLA_2_ enzymes have been reported [62], the secretory PLA_2_ (sPLA_2_ groups I, II, III, V, IX, and X with relatively small molecular weights) and the cytosolic PLA_2_ (cPLA_2_ groups IV, VI, VII, and VIII with higher molecular weights). Inhibition of specific PLA_2_ constitutes a potentially useful approach for treating a wide variety of inflammatory disorders such as spetic shock, adult respiratory distress syndrome, arthritis, and acute pancreatitis [61].

Petrosaspongiolides M–R (**46**–**50**) together with 21-hydroxy derivatives of petrosaspongiolides K (**44a**) and P (**48a**), and spongidines A–D (**51**–**54**) were tested on five different sPLA_2_s belonging to the groups I (*Naja naja* venom and porcine pancreatic enzymes), II (human synovial recombinant and rat air-pouch secretory enzymes), and III (bee venom enzyme) [60,61].

Among petrosaspongiolide derivatives, **46** and **48a** inhibited mainly human synovial PLA_2_ with IC_50_ values of 1.6 and 5.8 μM, respectively, compared to manoalide (**1**) (IC_50_ = 3.9 μM) [60,61]. Petrosaspongiolide M (**46**) also inhibited bee venom PLA_2_ enzyme with IC_50_ of 0.6 μM, compared to **1** (IC_50_ of 7.5 μM) [60].

The mechanism of action of petrosaspongiolides M–R (**46**–**50**) as anti-inflammatory marine metabolites has been the topic for many research articles [63–68]. The covalent binding of **46** to bee venom PLA_2_ has been investigated by mass spectrometry and molecular modeling. The mass increment observed was consistent with the formation of a Schiff base by reaction of a PLA_2_ amino group with the hemiacetal function at the C-25 atom of the petrosaspongiolide M *γ*-hydroxybutenolide ring [63]. The molecular mechanism of inactivating the bee venom and the human type IIA secretory PLA_2_s by petrosaspongiolides R (**50**) [67], and M (**46**) [68], respectively, has been investigated. In both cases, either covalent (imine formation) and/or non-covalent (van der Waals) interactions contributed to the inhibitory activity against PLA_2_ enzymes [67,68]. Due to potent anti-inflammatory properties of petrosaspongiolides, their chemical synthesis has been interestingly investigated. Recently, the first enantioselective synthesis of petrosaspongiolide R (**50**) has been successfully performed [69].

## 3. Triterpenes (C_30_)

Steroidal triterpenes were the first marine isoprenes to be discovered in the 1930s. Scientific interest has been driven towards these metabolites due to the isolation of biosynthetically unprecedented derivatives possessing a broad spectrum of bioactivity(ies). Marine triterpenoids have been reported from various marine macroorganisms. In this section, we survey two examples of triterpenoidal metabolites namely isomalabaricane triterpenes and steroidal saponins obtained from marine sponges with particular attention being drawn to their pharmacological significance.

### 3.1. Isomalabaricane triterpenes

Malabaricol (**55**) is the chief triterpene constituent of a yellow pigment obtained from the wood of the terrestrial plant *Ailanthus malabarica* (family Simaroubaceae), after which the whole group of related compounds was named [70–72]. Malabaricane, the trivial name of this group of compounds, was given to the hydrocarbon system (3S^*^,3aR^*^,5aS^*^,9aS^*^,9bS^*^)-3a,6,6,9a-tetramethyl-3-(1,5,9- trimethyldecyl)perhydr-obenz[*e*]indene, where the tricylic nucleus has a *trans*-*anti*-*trans* ring junction [71,72].

**Figure:**
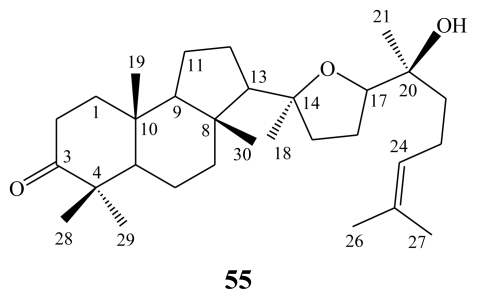


The malabaricanes are structurally characterized by a tricyclic triterpenoid core and a conjugated polyene side chain [70–72], whereas the isomalabaricane skeleton is embedded in a 4,4,8,10-tetramethyl-perhydrobenz[*e*]indene with a *trans*-*syn*-*trans* ring junction, that leads to an unfavorable twist-boat conformation for the central ring [73,74].

Isomalabaricane triterpenes were first reported from a Fijian collection of the sponge *Jaspis stellifera* [73] and the Somalian marine sponge *Stelletta* sp. [74]. Since then, they have been isolated from several genera of marine sponges belonging to the order Astrophorida including members of the genera *Rhabdastrella* [75,80,82,86,93,94,96,100], *Stelletta* [77–79,85,88,92], *Jaspis* [81,87,89,98,99,101,102], and *Geodia* [83,90,95].

Isomalabaricane triterpenoids having polyene conjugated functionality can be classified into three groups: (1) stelletins principally possessing the *γ*-pyrone functionality, which could be ring-opened in some of its congeners yielding the side chain with terminal free carboxylic acid and methyl moieties, (2) stelliferins oxygenated at C-22, and 3) globostellatic acids whose main feature is a carboxyl group at C-4. In addition to triterpenoids, the isomalabaricane core has been also recognized in some sesqui- and/or sesterterpenes. The isomalabaricane terpenoids were sometimes trivially named according to their sponge origin.

Upon light exposure, the isomalabaricane-type terpenes readily isomerize at the C-13 position. Therefore, during isolation and characterization processes, they rapidly equilibrate into a 1:1 mixture of the 13*E* and 13*Z* isomers [78–80,88,89,98,99]. Nevertheless, these compounds continue to gain a great deal of attention because of their significant cytotoxic activity [79,89], whereas the nature of the natural isomer, either 13*E* or 13*Z* or both, is still unresolved. Recently it was reported that the ^1^H NMR spectrum of a crude extract obtained from the fresh sponge *Rhabdastrella* aff. *distinca* (Hainan, the South China Sea) revealed that it mostly contained isomalabaricanes with the 13*E*-configuration (H-15 of most derivatives appeared around 7.0 ppm). Thus, the 13*Z* isomers were suggested in this case to be formed through isomerization during the isolation and analytical procedures [86].

Stelletins comprise the first group of isomalabaricane-type triterpenoids. Stelletin A (**56**) was recognized in 1981 as a yellow triterpenoidal pigment from the Fijian marine sponge *Jaspis stellifera* [73]. Later, it was obtained together with its *E* isomer, stelletin B (**57**), from the marine sponge *Stelletta tenuis* collected off Hainan Island, China [77]. Stelletin A (**56**) revealed significant cytotoxicity against murine leukemia P388 cell line with IC_50_ of 2.1 nM [77].

**Figure:**
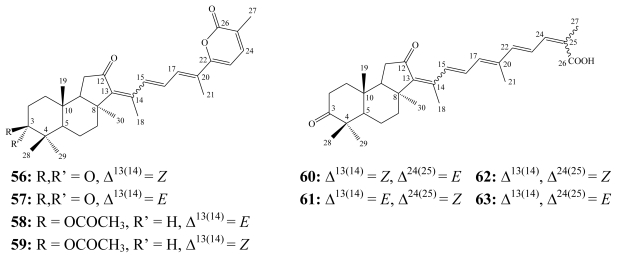


Stelletin G (**62**), with an opened *γ*-pyrone and featuring terminal -COOH and -CH_3_ functionalities, was isolated together with **56** from *J. stellifera* [73]. Later, stelletins G (**62**) was reported from the Australian marine sponge *Stelletta* sp. together with stelletins E (**60**) and F (**61**) [78]. The *E* isomer of stelletin G (**62**) was isolated from the marine sponge *Rhabdastrella globostellata* collected from the South China Sea and it was given the trivial name rhabdastrellic acid–A (**63**) [75,76].

Research interests have been intensively driven toward this group of triterpenoidal derivatives, which led to the isolation of eight further stelletins C,D, and H–M [78–80,82,85], in addition to 22,23-dihydrostelletin D [81].

Rhabdastrellins A–F (**64**–**69**), along with stelletins L (**70**) and M (**71**), were obtained from the marine sponge *Rhabdastrella* aff. *distinca* collected from a coral reef off Hainan, in the South China Sea [86]. Four of the rhabdastrellins (**64**–**67**) exhibited a primary alcohol moiety at C-29 instead of a methyl group as for the stelletins and the other two rhabdastrellins E (**68**) and F (**69**). While all rhabdastrellins and stelletins L and M share a hydroxyl group at C-3 instead of a carbonyl group as in other stelletins [86].

**Figure:**
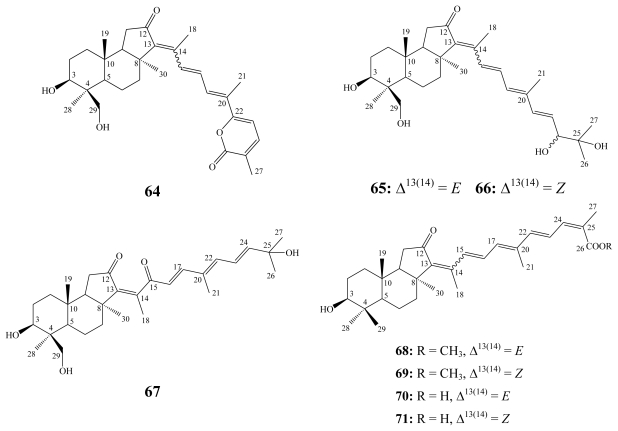


The antiproliferative profile of stelletins A–F (**56**–**61**) has been examined at the National Cancer Institute (NCI, Australia) against 60 cell lines. Due to the rapid isomerization upon light exposure, stelletins were tested as isomeric pairs. Stelletin C(**58**)/D(**59**) pair was the most potent derivative with a mean panel GI_50_ of 0.09 μM. The stelletin E(**60**)/F(**61**) pair was approximately 10-times less potent (mean GI_50_ of 0.98 μM) [79].

Apoptotic cell death is a stress response of cells to cytotoxic agents that might be executed either through a receptor-mediated pathway that activates caspase-8 or through a receptor-independent pathway that involves the cyclin-kinase inhibitors p53/p21. Both pathways lead to a translocation of pro-apoptotic Bax protein to the mitochondria, thereby resulting in a dissipation of mitochondrial membrane potential, activation of caspase-3, and execution of the apoptotic machinery [84].

Stelletin A (**56**) demonstrated a differential cytotoxicity against human leukemia HL-60 cells (IC_50_ 0.9 μM) compared to human prostate cancer LNCaP cells (IC_50_ 260 μM) by activation of NADPH oxidase, which induces oxidative cell death through a FasL–caspase-3-apoptotic pathway [83]. Stelletins B (**57**) and E (**60**) revealed selective cytotoxicity toward p21-deficient human colon tumor HCT-116 cells with IC_50_ values of 0.043 and 0.039 μM, respectively [80]. Stelletins L (**70**) and M (**71**) exhibited selective cytotoxicity against stomach cancer AGS cells with IC_50_ values of 3.9 and 2.1 μM, respectively [85]. Rhabdastrellic acid–A (**63**) also inhibited proliferation of human leukemia HL-60 cells with an IC_50_ value of 1.5 μM through inhibition of the PI3K/Akt pathway and induction of caspase-3 dependent-apoptosis [76]. Only rhabdastrellin A (**64**) possessed moderate inhibitory activity toward human leukemia HL-60 cells (IC_50_ = 8.7 μM) while other rhabdastrellins were inactive (IC_50_ > 20 μM) [86].

Stelliferins are the second group of isomalabaricane triterpenes. To the best of our knowledge, 13 compounds belonging to this group have been reported. In addition to stelliferins A–F (**72**–**77**), which have been isolated from the Okinawan marine sponge *Jaspis stellifera* [87], stelliferin G (**78**) and 29-hydroxy derivatives of stelliferins A (**79**) and E (**80**) have been isolated from an unidentified species of the genus *Jaspis* collected near Tonga [89].

**Figure:**
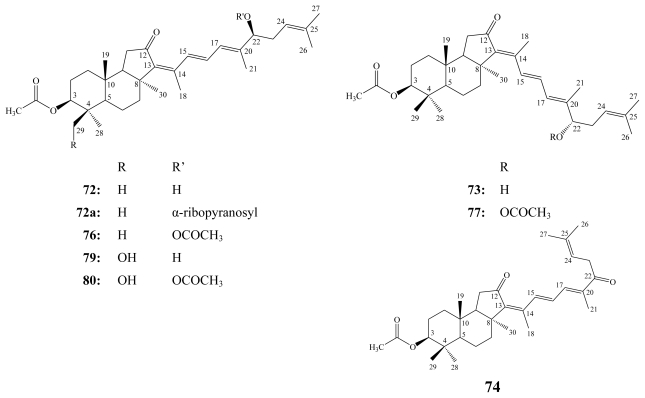


The 29-hydroxy derivative of stelliferin D (**81**) together with 3-epimeric isomers of **79** and **80** were reported from the marine sponge *Stelletta globostellata* collected by SCUBA off Mage-jima Island, Japan [88]. Whereas stelliferin riboside (**72a**), the first example of a glycosylated stelliferin, was isolated from the Fijian sponge *Geodia globostellata* [90].

**Figure:**
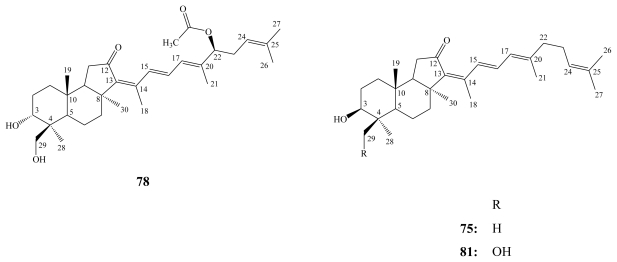


Stelliferins A–F (**72**–**77**) exhibited potent *in vitro* antineoplastic activities against murine lymphoma L1210 cells (IC_50_ of 1.1–5.0 μM) and human epidermoid carcinoma KB cells (IC_50_ of 2.8–13.0 μM) [87], while the isomeric mixture of stelliferin G (**78**) and 29-hydroxystelliferin A (**79**) showed the highest inhibitory activity against the melanoma MALME-3M cell line with IC_50_ values of 0.2 and 0.4 μM, respectively [89]. Stelliferin riboside (**72a**) displayed moderate cytotoxicity against ovarian A2780 cancer cells (IC_50_ = 60 μM) [90].

Due to the significant antiproliferative activity exhibited by stelletins and stelliferins, research efforts have been directed towards their chemical synthesis. In 1999, Raeppel *et al*. successfully synthesized the common *trans*-*syn*-*trans* perhydrobenz[e]indene moiety in the isomalabaricane-type terpenoids, which enabled the chemical synthesis of stelletins and stelliferins [91].

Globostellatic acid (**82**) is the prototype of the third group of isomalabaricane-type triterpenoids sharing carboxylation at C-4. It was first isolated together with three other derivatives, globostellatic acids B–D, from the marine sponge *Stelletta globostellata* collected off Mage Island near Kagoshima, Japan [92].

Other globostellatic acid congeners, F–M, and X methyl esters, have been reported from different collections of the Indonesian marine sponge *Rhabdastrella globostellata* [93,94].

Globostellatic acids revealed potent cytotoxicity similar to the stelletins and stelliferins. Globostellatic acids A–D demonstrated significant cytotoxicity against murine leukemia P388 cells with IC_50_ values of 0.2–0.8 μM [92].

For cytotoxicity toward mouse lymphoma L5178Y cells, the 3-*O*-deacetyl congeners, globostellatic acids H/I (**83**/**84**) were the most active with an IC_50_ of 0.31 nM. However, acetylation of the C-3 hydroxyl group decreases its bioactivity abruptly, as in globostellatic acids J/K (**85**/**86**), with an IC_50_ of 8.28 nM. The reverse was found for the 13*Z* isomer of stelliferin riboside (**72a**) that revealed higher activity than its 3-*O*-deacetyl congener with IC_50_ values of 0.22 and 2.40 nM, respectively [93].

On the other hand, globostellatic acids showed only moderate or no cytotoxicity against either human cervix carcinoma HeLa or rat pheochromocytoma PC-12 cell lines [93]. Two globostellatic acid X methyl esters (**87** and **88**), possessing the 13*E*-geometry, inhibited proliferation of human umbilical vein endothelial cells (HUVECs), 80- to 250-fold greater in comparison to several other cell lines and hence inhibiting angiogenesis which if pathologically uncontrolled, accompanies several diseases such as atherosclerosis, arthritis, diabetic retinopathy, and cancer.

**Figure:**
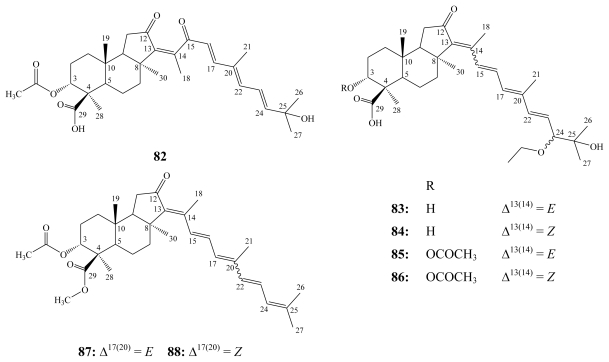


13*E*,17*E*- Globostellatic acid X methyl ester (**87**) also inhibited basic fibroblast growth factor (bFGF)-induced tubular formation and vascular endothelial growth factor (VEGF)-induced migration of HUVECs. In addition, **87** induced apoptosis of HUVECs without affecting their VEGF-induced phosphorylation of ERK1/2 kinases [94].

Geoditins, which are stelliferin-related isomalabaricane triterpenoids, are mainly oxygenated at both C-22 and C-25. Five geoditins (**89**–**93**) were obtained from the marine sponges *Geodia japonica* [95] and *Rhabdastrella* aff. *distinca* [96] collected at different locations in the South China Sea.

**Figure:**
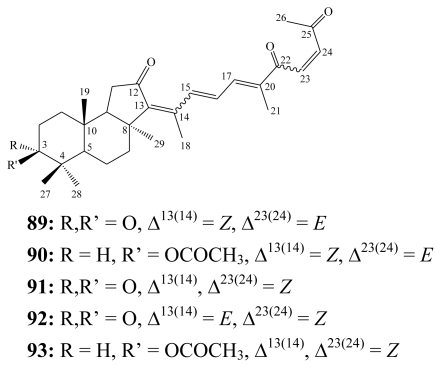


Geoditins (**89**–**93**) were submitted for bioassays against several human tumor cell lines including HL-60 (promyelocytic leukemia), PC-3MIE8 (prostate carcinoma), BGC-823 (gastric carcinoma), MDA-MB-423 (breast carcinoma), Bel-7402 (hepatocellular carcinoma) and HeLa (cervical carcinoma) cells. Isogeoditin A (**91**) showed significant cytotoxicity towards the former three cell lines with IC_50_ values of 0.3, 0.2 and 1.0 μM, respectively. While 13*E*-isogeoditin A (**92**) revealed no cytotoxic activity, implying that the *Z*-geometry at C-13 enhances antiproliferative activity compared to the *E*-form [96]. Geoditin A (**89**) proved to be cytotoxic against HL-60 cells [IC_50_ = 6.7 μM)] while geoditin B (**90**) exhibited relatively weak cytotoxicity. Mechanistically, geoditin A (**89**) markedly induced reactive oxygen species (ROS), decreased mitochondrial membrane potential and mediated a caspase-3 apoptosis pathway [97].

Jaspiferals (**94**–**103**) and aurorals (**104**–**107**) are isomalabaricane-type terpenoids differentiated into nortriterpenoids, norsesterterpenoids and norditerpenes possessing a 3*α*-hydroxy group. Jaspiferals A–G (**94**–**100**) were purified from the Okinawan marine sponge *Jaspis stellifera* [98] while the 3-*O*-acetyl and methyl ester derivatives of jaspiferals B (**101**), D (**102**) and E (**103**) were obtained from a new species of *Jaspis* collected at the Vanuatu Islands [99]. Aurorals (**104**–**107**) have been isolated from the New Caledonian marine sponge *Rhabdastrella globostellata* [100].

**Figure:**
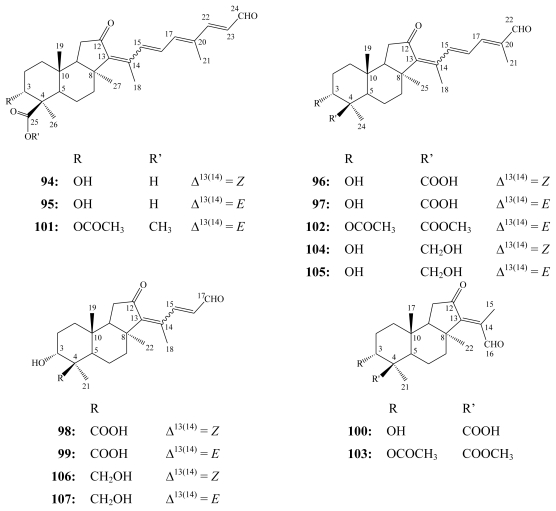


Jaspiferals A–G (**94**–**100**) exhibited *in vitro* cytotoxicity against murine lymphoma L1210 cells with IC_50_ values of 1.6–10.4 μM, whereas only jaspiferals E–G (**98**–**100**) revealed antineoplastic activity against human epidermoid carcinoma KB cells (IC_50_ of 5.2–14.7 μM) [98]. Jaspiferal G (**100**) exhibited antifungal activity against *Cryptococcus neoformans* (MIC, 144 μM) and *Trichophyton memtagrophytes* (MIC, 36 μM), and antibacterial activity against *Sarcina lutea* (MIC, 144 μM), while the mixture of jaspiferals E (**98**) and F (**99**) showed antifungal activity against *T. memtagrophyes* (MIC, 134 μM) [98]. On the other hand, the 3-*O*-acetyl, methyl ester derivatives of jaspiferals B (**101**), D (**102**) and E (**103**) revealed weak cytotoxicity against L1220 cells (IC_50_ > 8.8 μM) [99].

Aurorals (**104**–**107**), which differ from jaspiferals C–F (**96**–**99**) by the presence of a primary alcohol group at the C-4 position, exhibited stronger cytotoxicity against KB cells. The isomeric mixtures of aurorals (**104**/**105**), (**106**/**107**) and jaspiferals C/D (**96**/**97**) showed IC_50_ values of 0.5, 22.2 and 13.3 μM, respectively, while jaspiferals E/F (**98**/**99**) were inactive up to 27 μM [100].

Jaspolides represent another example of isomalabaricane-type terpenoids of either monomeric or dimeric congeners. Monomeric congeners of jaspolides could be classified into triterpenes, jaspolides A (**108**) and B (**109**); sesterterpene, jaspolide F (**113**); diterpenes, jaspolides C (**110**) and D (**111**); and nortriterpene, jaspolide E (**112**) which were all isolated from the marine sponge *Jaspis* sp. collected from the South China Sea [101].

**Figure 2:**
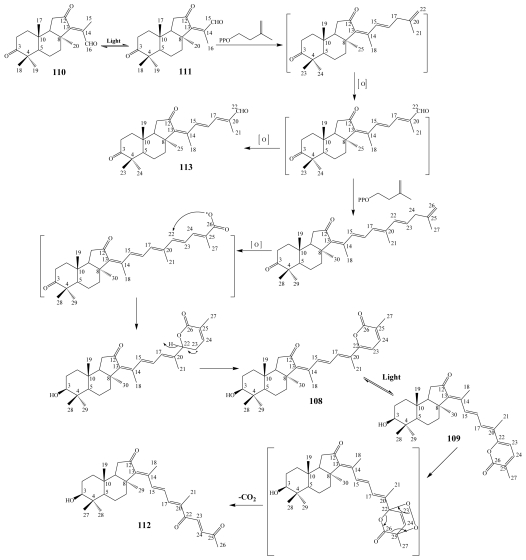
Proposed biogenetic transformation of jaspolides A–F (**108**–**113**) [101].

**Figure 3:**
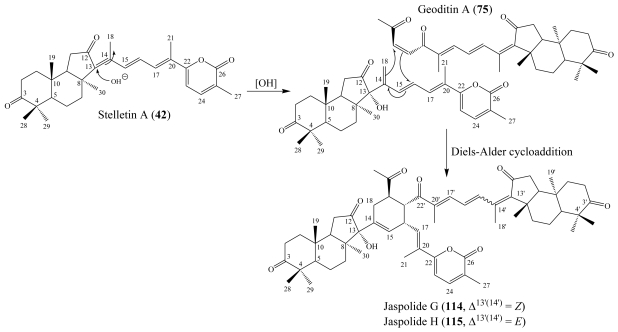
Postulated biogenetic pathway of jaspolides G (**114**) and H (**115**) [102].

A presumable biogenetic transformation scheme of jaspolides A–F (**108**–**113**) (Figure 2), revealed that light-induced isomerization is responsible for the jaspolides A/B (**108**/**109**) and C/D (**110**/**111**) isomeric pairs. In addition it substantiated jaspolide D (**111**) as a precursor to jaspolide F (**113**), formed through condensation with an isoprenyl pyrophosphate (IPP) followed by oxidation at a terminal methyl group [101]. Jaspolides G (**114**) and H (**115**) are dimeric isomalabaricane congeners which were isolated from the same Chinese sponge *Jaspis* sp. and their proposed biogenetic pathway (Figure 3) suggested that they were derived from stelletin A (**56**) yielding the left moiety, and the nortriterpene, geoditin A (**89**) yielding the right moiety [102].

Jaspolide B (**109**) arrested HL-60 cells in the G_2_/M phase of the cell cycle and induced apoptosis in a dose- and time-dependent manner. Jaspolide B with an IC_50_ value of 0.61 μM exhibited a comparable efficacy as that of paclitaxel (IC_50_ = 0.78 μM). These results suggested **109** to be a promising anticancer agent for chemotherapy of leukemia by prohibiting cell cycle progression at the G_2_/M phase and triggering apoptosis [103].

In a further study with human hepatoma cells, jaspolide B (**109**) inhibited the growth of Bel-7402 and HepG2 cells with IC_50_ values of 29.1 and 29.5 μM, respectively. Incubation with 0.5 μM of **109** caused time-dependent induction of apoptosis in Bel-7402 as confirmed by the enhancement of mitochondrial masses, cell membrane permeability, and nuclear condensation. In conclusion, the anticancer effect of jaspolide B involves multiple mechanisms including apoptosis induction, cell cycle arrest, and microtubule disassembly but these were weaker than colchicine, a well-known microtubule disassembly agent [104]. These multiple mechanisms of jaspolide B, especially the apoptosis induction, pose interesting perspectives for further exploration of the isomalabaricane-type terpenes as potential anticancer agents.

Since the class of isomalabaricane terpenoidal metabolites has been reported in the literature from different sponge species of the genera *Rhabdastrella*, *Stelletta*, *Jaspis*, and *Geodia* as shown above, the identity of these sponges has been questioned and reevaluated. Interestingly, the taxonomic reevaluation of these sponges revealed that they all might be reassigned to *Rhabdastrella globostellata* (class Demospongiae; order Astrophorida; family Ancorinidae) [80]. However, it could not be ascertained for the isomalabaricane producing *Stelletta* sp. from Somalia [74] and *Stelletta tenuis* from China [77]. The latter, collected from an identical location (Hainan Island), was taxonomically recognized as *R. globostellata* [75].

### 3.2. Steroidal saponins

In the Kingdom Animalia, steroidal and triterpene glycosides are predominant metabolites of starfishes and sea cucumbers, respectively [108]. In addition, these types of glycosides have also been isolated from marine sponges. To the best of our knowledge, around 80 sponge triterpenoidal glycosides have been reported to date, including erylosides [107–114], formosides [115,116], nobiloside [117], and sokodosides [118] from different sponge species of the genus *Erylus*; sarasinosides from the marine sponges *Asteropus sarasinosum* [120–123], *Melophlus isis* [124], and *M. sarassinorum* [125]; mycalosides from *Mycale laxissima* [126–128]; ectyoplasides and feroxosides from the Caribbean marine sponge *Ectyoplasia ferox* [129,130]; ulososides from *Ulosa* sp. [131,132]; wondosterols from a two-sponge association [133]; and pachastrelloside A from a marine sponge of the genus *Pachastrella* [134]. The majority of these glycosides belong to norlanostane-triterpenoidal saponins, derived from lanosterol or related triterpenes as a result of oxidative elimination of one or two methyl groups.

Penasterol (**116**), an acidic steroidal metabolite closely related to lanosterol (**117**) and possessing potent antileukemic activity, was originally isolated from the Okinawan marine sponge *Penares* sp. in 1988 [105]. Penasterol together with its analogs penasterone and acetylpenasterol, isolated from the Okinawan marine sponge *Penares incrustans*, inhibit IgE-dependent histamine release from rat mast cells [106].

Eryloside A (**118**) was the first eryloside congener isolated from the Red Sea sponge *Erylus lendenfeldi* (class Demospongiae; order Choristida; family Geodiidae) [107]. Twenty eight additional erylosides (A–F, F_1_–F_7_, G–V) have been reported from different species of the genus *Erylus* including *E. goffrilleri* [109,114], *E. formosus* [110,113], *E. nobilis* [111], in addition to another collection of *E. lendenfeldi* [112].

**Figure:**
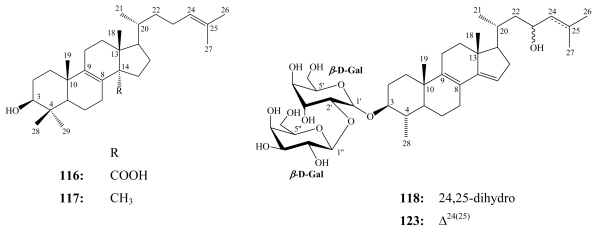


For eryloside A (**118**), antitumor activity against murine leukemia P388 cells with an IC_50_ = 5.7 μM and antifungal activity against *Candida albicans* (MIC = 21.1 μM) have been reported [107]. Eryloside E (**119**), glycosylated at C-30 through an ester linkage with the rare *t*-butyl substitution of the side chain, was isolated from an Atlantic sponge *Erylus goffrilleri* [109]. It revealed immunosuppressive activity with an EC_50_ of 1.8 μM and a therapeutic index (TI) of 9.5, which indicated that the immunosuppressive effect is specific and is not due to a general cytotoxic effect [109].

**Figure:**
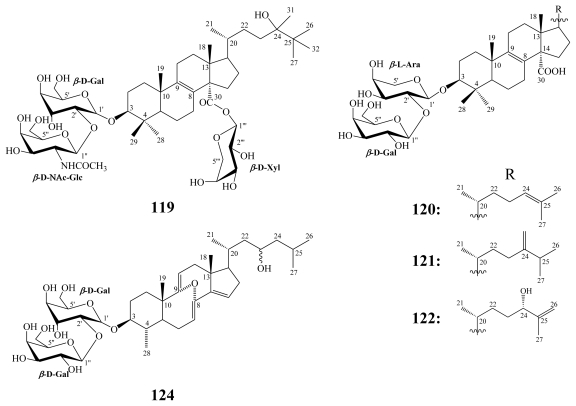


Eryloside F (**120**) was reported from two collections of the marine sponge *E. formosus* [110] and exhibited potent thrombin receptor antagonistic activity. Furthermore, it inhibited platelet aggregation *in vitro*. Against hepytocyte HepG2 cells, **120** possessed little activity [110]. Erylosides F_1_ (**121**) and F_3_ (**122**) were isolated along with nine other congeners from the Caribbean sponge *E. formosus* [113]. In contrast to its 24-epimer, eryloside F_3_ (**122**) induced early apoptosis in Ehrlich carcinoma cells at 130 μM, while erylosides F (**120**) and F_1_ (**121**) activated the Ca^2+^ influx into mouse spleenocytes at the same doses [113].

Erylosides K (**123**) and L (**124**) have been obtained together with **118** from another collection of the Red Sea marine sponge *Erylus lendenfeldi* [112]. While **123** was identified as the 24,25-didehydro congener of eryloside A, eryloside L (**124**) incorporated a naturally unprecedented 8*α*,9*α*-epoxy-4*α*-methyl-8,9-secocholesta-7,9(11),14-triene skeleton [112]. Erylosides A (**118**) and K (**123**) led to a 50% mortality rate in the brine shrimp assay at a concentration of 0.14 mM. Whereas, eryloside L (**124**) was inactive at the same concentration [112].

In addition to erylosides, the marine sponges *E. formosus* and *E. nobilis* produced other steroidal saponins identified as formosides A (**125**) [115] and B (**126**) [116]; and nobiloside (**127**) [117], respectively, whilst sokodosides A (**128**) and B (**129**) have been obtained from the marine sponge *Erylus placenta* [118]. A convergent synthesis of the trisaccharides of **129** has been successfully performed [119].

Formoside A (**125**) was first reported by Jaspars and Crews in 1994 from the Caribbean marine sponge *Erylus formosus* [115]. Later, it was isolated together with formoside B (**126**) from another collection of the same sponge from the Bahamas [116]. Formoside A (**125**) and its *N*-acetyl galactosamine derivative, formoside B (**126**) possess deterrent properties against predatory fish. Therefore, they were suggested to have important ecological functions, resembling those ascribed to similar compounds present in sea stars, sea cucumbers, and terrestrial plants [116].

**Figure:**
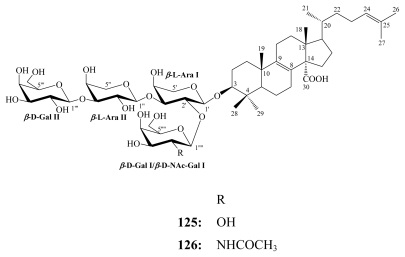


Nobiloside (**127**), a penasterol saponin, was reported from the marine sponge *E. nobilis* collected off Shikine-jima Island, Japan [117] and revealed the presence of a carboxylic group at C-30 in addition to uronic acid moieties. Nobiloside (**127**) inhibited neuraminidase from the bacterium *Clostridium perfrigens* with an IC_50_ of 0.5 μM [117].

**Figure:**
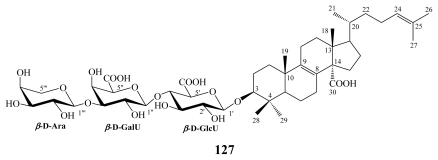


Sokodosides A (**128**) and B (**129**) were obtained from the marine sponge *E. placenta* collected off Hachijo Island, Japan [118]. They possessed a novel carbon skeleton as characterized by the presence of a combination of an isopropyl side chain and the 4,4-dimethyl steroid nucleus. Moreover, sokodoside B (**129**) exhibited double bonds at unusual positions Δ^8(9),14(15),16(17)^.

**Figure:**
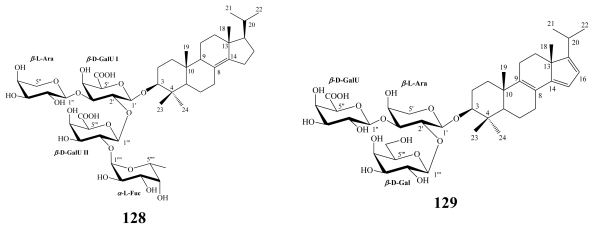


Both sokodosides displayed moderate antifungal activity against the fungus *Mortierella ramanniana* and the yeast *Saccharomyces cereivisiae,* but no antibacterial activity was found. Additionally, sokodosides A (**128**) and B (**129**) exhibited cytotoxic activity against P388 cells with IC_50_ values of 103 and 62 μM, respectively [118].

Sarasinosides follow erylosides in the number of isolated metabolites. To date, 21 sarasinoside congeners have been reported, which all featured a carbonyl group at C-23 position. Sarasinoside A_1_ (**130**) was the first steroidal saponin reported in the literature, even before eryloside A (**118**), from the Palauan marine sponge *Asteropus sarasinsum*, together with other eight new congeners [120–122]. Then, from the same sponge collected in the Solomon Islands, four additional sarasinosides D–G were reported [123]. From each of the marine sponges *Melophlus isis* (Guam) [124] and *M. sarassinorum* (Indonesia) [125], four sarasinoside congeners were isolated.

Among the sarasinoside congeners known to date, sarasinoside A_1_ (**130**) and B_1_ (**131**) exhibited piscicidal activity against *Poecilia reticulata* with LD_50_ values (48 h) of 0.3 and 0.6 μM, respectively [120,122].

**Figure:**
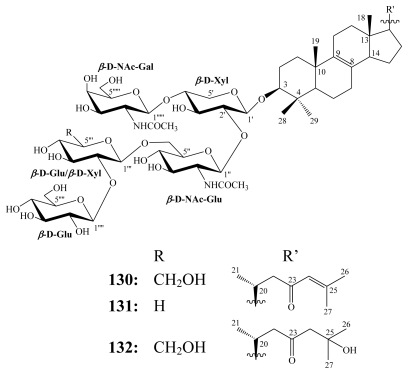


Sarasinoside A_1_ is known to possess moderate cytotoxicity *in vitro* against leukemia P388 [121] and K562 [124] cell lines with IC_50_ values of 2.2 and 5.0 μM, respectively. Sarasinoside A_3_, which differs from A_1_ (**130**) in having Δ^8(9),14(15)^ instead of Δ^8(9)^ unsaturation, exhibited mild cytotoxic activity with an IC_50_ of 13.3 μM [124].

In the agar diffusion antimicrobial assay (10 *μ*g/disc), sarasinoside A_1_ showed strong and selective activity against the yeast *S. cerevisiae* but was inactive against *B. subtilis* and *E. coli*. On the other hand, sarasinoside J (**132**) was active against *S. cerevisiae* and showed moderate antibacterial activity against *B. subtilis* and *E. coli* [125].

Mycalosides include eleven steroidal saponin congeners that were isolated from the Caribbean marine sponge *Mycale laxissima* (class Demospongiae; order Poecilosclerida; family Mycalidae) collected near San-Felipe Island, Cuba [126–128]. They were all characterized by having oxygenated C-4, C-15, and C-21 positions.

Mycaloside A (**133**) and G (**134**) as well as the total glycoside fraction did not influence nonfertilized eggs and the developing embryo up to the 8-blastomere stage at concentrations of up to 94.6 μM. However, these compounds were effective as spermatostatics when preincubated for 15 min with sea urchin sperm with an EC_50_ of 3.04 μM. The total glycoside fraction generated a less toxic effect (EC_50_ = 7.03 *μ*g/mL) [127].

**Figure:**
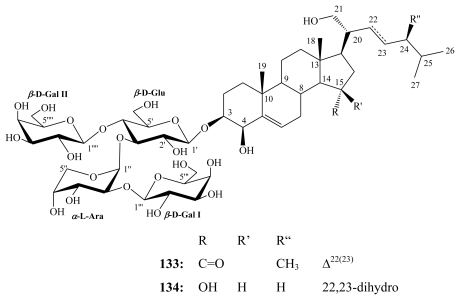


Ectyoplasides A (**135**) and B (**136**) were first isolated from the Caribbean sponge *E. ferox* (class Demospongiae; order Axinellida; family Raspaliidae) collected along the coasts of San Salvador Island, Bahamas [129]. The compounds are C-4 norpenasterol triterpenoidal derivatives. Later, ectyoplasides were reisolated together with feroxosides A (**137**) and B (**138**) from the same sponge collected along the coasts of Grand Bahama Island [130]. Feroxosides have been shown to be unusual C-4 norlanostane triterpenes glycosylated with a rhamnose-containing tetrasaccharide chain.

**Figure:**
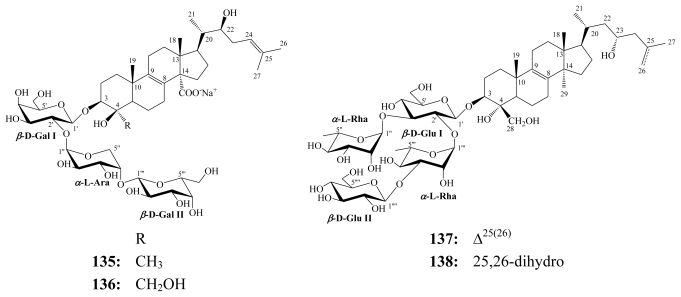


Against murine fibrosarcoma WEHI164, murine leukemia P388, and murine monocyte-macrophage J774 cell lines, both ectyoplasides (**135** and **136**) exhibited moderate *in vitro* cytotoxic activity with IC_50_ values ranging from 9.0 to 11.4 μM [129], whilst against the latter cell line, feroxosides (**137** and **138**) were mildly cytotoxic (IC_50_ = 17.6 μM) [130].

Pachastrelloside A (**139**) was obtained from the marine sponge *Pachastrella* sp. (Kagami Bay, Japan) and revealed the presence of a cholest-5,24-diene-2*α*,3*β*,4*β*,7*α*-tetraol aglycone that was glycosylated at the C-4 and C-7 positions with *β*-D-xylopyranose and *β*-D-galactopyranose moieties, respectively [134].

**Figure:**
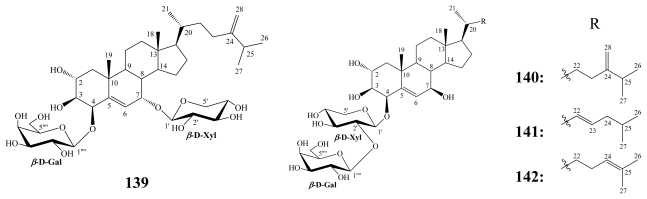


A Korean sponge-association composed of *Poecillastra wondoensis* and *Jaspis wondoensis* resulted in the isolation of wondosterols A–C (**140**–**142**), which are structurally related to **139** [133]. Wondosterols were shown to have a *β*-OH group at C-7 and they were all diglycosylated at C-3 with *β*-D-xylopyranose connected to *β*-D-galactopyranose.

Wondosterols A–C (**140**–**142**) were weakly cytotoxic against P388 cells (IC_50_ = 63 μM) and at a concentration of 10 *μ*g/disk only **140** and **142** showed antibacterial activities against *P. aeruginosa* and *E. coli* [133]. Pachastrelloside A (**139**) inhibited cell division of fertilized starfish (*Asterina pectinifera*) eggs at 35 μM [134].

## 4. Future Aspects

The enormous diversity of marine natural products combined with improved global concerns to find new therapeutic agents for the treatment of different ailments provide the stimulus to evaluate marine natural products in clinical trials. Marine drug discovery faces many obstacles including a sufficient supply, and the low concentrations of some compounds that may account for less than 10^−6^% of the wet weight [135]. However, there have been substantial advances, suggesting that sustainable sourcing could be achievable. Since the continuous and exhaustive harvesting of terrestrial drug lead resources proved to be unreliable and resulted in the frequent re-isolation of known compounds, researchers from academia and from pharmaceutical companies alike are now turning their focus to the sea in search for new lead structures from nature. Nevertheless, the large scale production of marine natural products for clinical use is a real challenge, and therefore environmentally sound and economically feasible alternatives are required.

Chemical synthesis is among the first strategies to be explored, but unfortunately the structural complexity of marine metabolites with novel mechanisms of action and high selectivity has resulted in only a few successful examples with this strategy such as the conus toxin ziconotide [136]. A second strategy, but also as labor-intensive, is to study the pharmacological significance of marine natural product pharmacophores and then attempt to define the critical pharmacophore that can result in practical drugs based on a marine prototype via chemical synthesis, degradation, modification or a combination of these.

Aquaculture of the source organisms, including sponges, tunicates, and bryozoans, with an aim at securing a sustainable supply of the active constituent(s), has progressed notably in cancer applications. However, in most cases the biomass currently generated is still far from that required, should a marine-based drug finally enter the pharmaceutical market [137]. Furthermore, the cultivation of invertebrates in their natural environment is subject to several hazards and threats, such as destruction by storms or diseases. An intriguing strategy has been to identify the true producers of bioactive compounds and to explore whether or not they are of microbial origin including bacteria, cyanobacteria, or fungi that are known to harbour within the tissues of marine invertebrates.

If bacterial or other associated microorganisms proved to produce the compounds of interest, a careful design of special culture media would be crucial for large-scale fermentation e.g., ET-743 production. Currently, only 5% or less of the symbiotic bacteria present in marine specimens can be cultivated under standard conditions [138]. Consequently, molecular approaches offer particularly promising alternatives through the transfer of biosynthetic gene clusters to a vector suitable for large-scale fermentation, thereby avoiding the obstacles in culturing symbiotic bacteria.

Oceans will play a potential role in the future to control and relieve the global disease burden. In spite of the substantial development that has been achieved in disclosing novel drug leads from marine resources, more efforts are still required for more chemical entities to reach to clinical applications.
